# Detecting Key Factors of Grasshopper Occurrence in Typical Steppe and Meadow Steppe by Integrating Machine Learning Model and Remote Sensing Data

**DOI:** 10.3390/insects13100894

**Published:** 2022-09-30

**Authors:** Longhui Lu, Weiping Kong, Eerdengqimuge, Huichun Ye, Zhongxiang Sun, Ning Wang, Bobo Du, Yantao Zhou, Weijun, Wenjiang Huang

**Affiliations:** 1 Key Laboratory of Digital Earth Science, Aerospace Information Research Institute, Chinese Academy of Sciences, Beijing 100094, China; 2 International Research Center of Big Data for Sustainable Development Goals, Beijing 100094, China; 3 Key Laboratory of Quantitative Remote Sensing Information Technology, Aerospace Information Research Institute, Chinese Academy of Sciences, Beijing 100094, China; 4 Grassland Workstation of Xilinguole League, Xilingol 026000, China; 5 China Agricultural Museum, Beijing 100125, China; 6 Institute of Grassland Research, Chinese Academy of Agricultural Sciences, Hohhot 010010, China; 7 Center for Biological Disaster Prevention and Control, National Forestry and Grassland Administration, Shenyang 110034, China; 8 Hulun Buir Forestry and Grassland Development Center, Hulun Buir 021008, China; 9 University of Chinese Academy of Sciences, Beijing 100049, China

**Keywords:** grasshopper, typical steppe, meadow steppe, maxent, remote sensing

## Abstract

**Simple Summary:**

Grasshoppers are among the most dangerous agricultural pests of China. However, the monitoring, prediction and control of grasshoppers are complex and difficult. Therefore, it is crucial to detect the key factors affecting the spatial distribution of grasshopper occurrence, understand the role of the environmental factors in grasshopper occurrence, and study whether different laws exist between different grass types. Here we conduct a species–environmental matching model integrated by Maxent model and remote sensing data to identify the spatial distribution of grasshopper occurrence in Inner Mongolia of China, analyze the related environmental variables and detect the most relevant environmental factors for grasshopper occurrence both in typical steppe and meadow steppe.

**Abstract:**

Grasshoppers mainly threaten natural grassland vegetation and crops. Therefore, it is of great significance to understand the relationship between environmental factors and grasshopper occurrence. This paper studies the spatial distribution and key factors of grasshopper occurrence in two grass types by integrating a machine learning model (Maxent) and remote sensing data within the major grasshopper occurrence areas of Inner Mongolia, China. The modelling results demonstrate that the typical steppe has larger suitable area and more proportion for grasshopper living than meadow steppe. The soil type, above biomass, altitude and temperature mainly determine the grasshopper occurrence in typical steppe and meadow steppe. However, the contribution of these factors in the two grass types is significantly different. In addition, related vegetation and meteorological factors affect the different growing stages of grasshoppers between the two grass types. This study clearly defines the different effects of key environmental factors (meteorology, vegetation, soil and topography) for grasshopper occurrence in typical steppe and meadow steppe. It also provides a methodology to guide early warning and precautions for grasshopper pest prevention. The findings of this study will be helpful for future management measures, to ensure grass ecological environment security and the sustainable development of grassland.

## 1. Introduction

Grasshoppers are among the most dangerous agricultural pests, and their control is critical to food security [1]. Grasshoppers include different species, and they are also the main grass pests in China [2]. Grasshoppers threaten the natural grassland vegetation and crops [3,4]. When an outbreak of grasshoppers happens, it causes serious environmental and socio-economic consequences [2,5], such as grassland degradation and desertification, further exacerbating the deterioration of grassland environments and ecosystems [6]. Significant economic damage can occur in the areas of agriculture and husbandry also.

At present, China’s natural grass area is the largest in the world. Based on the inventory data, grassland area damaged by grasshoppers in China generally exceeds more than 6.67 million hectares every year, with the highest reported loss of 13.33 million hectares in 2009. China has also gradually strengthened the prevention and control measures against grasshoppers [7]. However, climate change [8], human activities in grassland utilization [9], vast areas and diverse types of grass [10], and the long survival time of grasshoppers’ eggs [11] lead to the outbreak of grasshoppers once the environmental conditions are suitable. This makes the monitoring, prediction and control of grasshoppers complex and difficult. Therefore, it is crucial to determine the key factors affecting the spatial distribution of grasshopper occurrence, understand the role of environmental factors in grasshopper occurrence, and study whether different rules exist between different grass types.

Recently, several studies showed that grasshopper occurrence is affected by different environmental factors or geographical attributes [12,13,14,15,16,17,18,19,20,21,22,23,24,25,26,27,28,29,30,31,32,33,34,35,36,37,38]. For instance, studies focusing on single factors influencing grasshopper occurrence demonstrate that grasshopper occurrence has a high correlation with plants [13]. Grasshopper occurrence and spatial distribution strongly respond to local vegetation height and amount of bare ground [14]. In addition, related studies demonstrate that microhabitat, including plant structure, vegetation cover and community composition, have repercussions for grasshopper occurrence [15,16,17]. Other studies focusing on soil attributes show that changes in soil temperature and moisture, especially extremes in temperature and moisture, have a substantial effect on grasshopper occurrence by affecting the eggs’ development, survival and hatching [10,18,19]. The soil type, as well as topsoil moisture content can affect grasshopper occurrence by influencing grasshopper density [18,20]. Moreover, the region-specific topography, mainly including altitude and slope, has clear effects on the distribution of grasshopper occurrence [13,21,22,23,24,25]. Several studies on the relationship between the microclimate (mainly including temperature and precipitation) and the distribution of grasshopper occurrence [9,17,25,26,27,28,29,30] exist. The temperature affects grasshopper occurrence by affecting the development, survival and reproduction of grasshoppers [31], due to the fact that warming accelerates the incubation of grasshopper eggs and reduces drought stress [32]. Early summer drought increases the survival of grasshoppers [33]. Temperature changes also affect the availability of suitable grasshopper habitats and cause grasshopper assemblage shifts [34]. Precipitation mainly affects grasshopper occurrence by affecting the soil moisture [20]. Furthermore, studies focusing on multiple factors affecting grasshopper occurrence [35,36] exist. For instance, researchers have analyzed grasshopper occurrence by integrating the elevation, slope, soil type, sand content and coverage conditions [5], coupling the vegetation heterogeneity, altitude and cover of bare ground [37], and combing the soil conditions, climate and plant nutrient content [38]. All these studies illustrate a considerable range of habitat preferences for the grasshoppers [39]. Therefore, it is crucial to find the relationship between grasshopper occurrence and the region-special environment factors [40,41].

**Figure 1:**
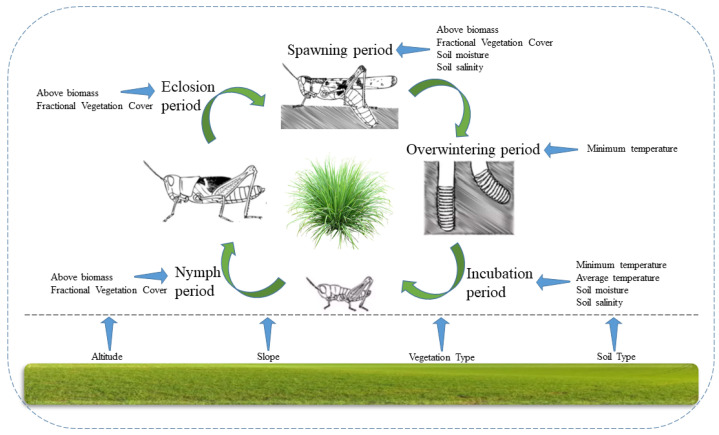
The environmental factors theoretically affecting the grasshopper occurrence. Altitude, slope, vegetation type and soil type are static geographical factors which affect all the spawning, overwintering, incubation, nymph and eclosion periods. Temperature, vegetation condition and soil condition are dynamic meteorological, vegetation and soil factors which have diverse impacts in different grasshoppers’ life cycle.

According to a large number of studies and statistical results on the physiology and life cycle of grasshopper, grasshopper occurrence shows the characteristics presented in Figure 1. Grasshoppers are suitable for living in specific geographical areas, such as a certain range of altitude and slope, and prefer certain vegetation types and soil types. Grasshoppers in Inner Mongolia grassland only have one generation per year [11]. *Oedaleus decorus asiaticus* Bei-Bienko is the dominant grasshopper species. Its whole life cycle is divided into spawning, overwintering, incubation, nymph and eclosion periods. During the spawning period, vegetation condition such as above biomass and vegetation cover, and soil moisture and soil salt, affect the selection of spawning sites. In the overwintering period, which can be almost half a year, extreme low temperature has a substantial effect causing the freezing of grasshoppers’ eggs and therefore leading to death [19]. In the incubation period, besides the extreme low temperature causing the death of grasshoppers’ eggs or causing diapause status [11], accumulated temperature, soil moisture and soil salt affect the success of incubation. In the nymph and eclosion periods, whether there is enough food and proper shelter, determines whether the nymph can normally grow.

Satellite-based remote sensing technology can provide dynamic and real-time observations, which allows grasshopper occurrence to be monitored [7]. The current study aims to predict grasshopper occurrence based on remote sensing and GIS using the NDVI [14,42], spectral analysis technique [41], land use and cover, soil moisture, precipitation [25], several essential ecological factors (such as vegetation condition, soil moisture and land surface temperature) [2], vegetation index [25,43], and microwave remote sensing [44]. By integrating GIS, remote sensing can be extensively applied in grasshopper occurrence monitoring [2].

Maximum entropy species distribution modeling (Maxent) was first used to simulate the geographical distribution of species [45]. It is a general machine learning algorithm [46], suitable for different species such as birds, terrestrial plants and bats [47,48,49], for example. However, the implication of Maxent in grasshopper occurrence is not well-documented. Currently, Maxent is the best-performing model for modeling species niche distribution, species occurrence and analyzing the relationship between species distribution and environmental factors [50,51].

In this study, the integration of the Maxent model and remote sensing data is used to conduct a species–environmental matching model. This study aims to: (1) identify the spatial distribution of grasshopper occurrence (the major grasshopper species: *Oedaleus decorus asiaticus* Bei-Bienko) in the major grasshopper occurrence areas of Inner Mongolia, which has the largest grass area of China; (2) analyze the related environmental variables and determine the most relevant environmental factors for grasshopper occurrence in typical steppe and meadow steppe; (3) find the different response of top predictor environmental variables in the grasshopper occurrence for typical steppe and meadow steppe. Using the Maxent model and remote sensing data to detect the key factors of grasshopper occurrence in two main grass types, a methodological support can be developed to guide early warning and related precautions for grasshopper pest prevention and control, which can be helpful for grass ecological environment security and sustainable husbandry development.

## 2. Materials and Methods

### 2.1. Study Area

**Figure 2:**
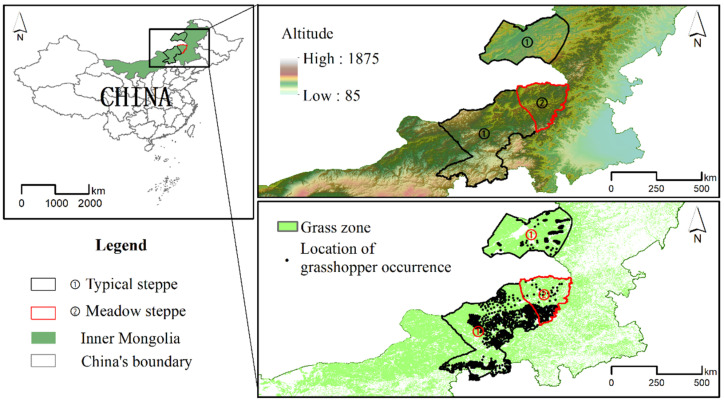
Study area with typical ① and meadow ② steppes represents two main grasshopper occurrence areas in the Inner Mongolia province of China. Altitude in the study area ranges from 500 m to 1800 m. Locations of grasshopper occurrence in the grass zone are shown by points.

The Inner Mongolia Autonomous Region has the largest grass area of China [52], which contains different grass types and has large grasshopper outbreaks every year [19]. The study area covers most of the grasshopper occurrence area of Inner Mongolia, which is located in Hulunbuir city and Xilingol league (cf. Figure 2). Hulunbuir and Xilingol also have mainly typical meadow steppeland and temperate grassland. The climate types within the study area are temperate continental climate, characterized by being arid with sparse precipitation and a decreasing precipitation trend from east to west. The main geomorphological types are plateau. The vegetation types are mainly *Stipa krylovii* Roshev grass, *Leymus chinensis* (Trin. ex Bunge) Tzvelev grass and weedy grass. The soil types mainly include meadow soils, Chernozems and Kastanozems. Different grasshopper species exist in the study area. *Oedaleus decorus asiaticus* Bei-Bienko and *Dasyhippus barbipes* Fischer von Waldheim are the main two grasshopper species. In Figure 1, area ① denotes the grasshopper occurrence area in the typical steppe of Hulunbuir and Xilingol, while area ② represents the grasshopper occurrence area in the meadow steppe of Xilingol.

### 2.2. Investigative Grasshopper Occurrence Data

In order to prevent outbreaks and control grasshopper populations, grassland pest control stations are located at the county level and are responsible for monitoring and reporting the area of grassland damaged by grasshoppers. The investigative grasshopper data of 2020 for the two grasshopper occurrence areas were obtained from the grassland pest control stations of Hulunbuir city and Xilingol league. The number of observations was 1113 in typical steppe and was 162 in meadow steppe. A regional survey method according to the standard in agricultural industry of People’s Republic of China (NY/T 1578-2007, Rules for investing locality and grasshopper in grassland) was used to investigate the overall grasshopper occurrence. The multi-point survey was carried out along the setting route that covered all the main natural geomorphic units, regular grasshopper occurrence areas and occasional grasshopper occurrence areas. Sampling plots were set at an average interval of 10 km, and then sampling points were set at an average interval of 100 m in each sample plot with repeated sampling for three times. The records consist of the occurrence area size and location (cf. Figure 1). In order to reduce the spatial autocorrelations, the grassland data were spatially rarefied with a radius of 1 km using SDM toolbox 2.0 (Python-based GIS toolkit for species distribution model analyses), and the observational grasshopper occurrence data were randomly assigned to the imaged grassland. Precise geographic grassland data were obtained from land cover data of 2020, which were downloaded from Resource and Environment Science and Data Center (https://www.resdc.cn/, accessed on 10 October 2020).

### 2.3. Environmental Factors Based on Remote Sensing Data

Based on the theoretical analysis of the factors affecting grasshopper occurrence (Figure 1), four types of environmental variables are used to simulate the suitability for grasshopper occurrence including the topography, meteorology, vegetation and soil indicators (cf. Table 1). In total, 16 environmental variables were extracted. The topographic indicators include the altitude and slope with a spatial resolution of 90 m. The meteorological indicators include the minimum and mean land surface temperature with a spatial resolution of 1 km. Mean minimum land surface temperature from August 2019 to May 2020 covering the spawning, overwintering, incubation and nymphal periods of grasshopper development was extracted. The mean land surface temperatures from April to May 2020 were calculated, covering the incubation period of the grasshoppers. The vegetation indicators include the vegetation type, above biomass and fractional vegetation cover with a spatial resolution of 1 km. The mean biomass and fractional vegetation cover from June to July 2020 were calculated, covering the nymphal period and eclosion period of the grasshoppers. The soil indicators include the soil type, soil moisture index and soil salinity index with a spatial resolution of 1 km. Mean soil moisture index and soil salinity index from August to October 2019 and April to May 2020 were calculated, covering the spawning period and incubation period of the grasshoppers.

### 2.4. Machine Learning Model (Maxent Model) and Validation

The machine learning model, Maximum entropy species distribution modeling (Maxent), downloaded from the American Museum of Natural History (version 3.4.1, https://biodiversityinformatics.amnh.org/open_source/maxent/, accessed on 1 September 2020 [45]), was used to model the spatial distribution of grasshopper occurrence and study the relevant environmental factors. Maxent has an efficient predictive performance for modeling species distributions and analyzing their relationship with environmental variables, using small or large sample sizes [46,53]. The Maxent model formula is given by [53]:

(1) $$P_{w}\left(y|x\right)=\frac{1}{Z_{w}\left(x\right)}exp\left(\overset{n}{\underset{i=1}{\sum }}w_{i}f_{i}\left(x,y\right)\right)$$

(2) $$Z_{w}\left(x\right)=\underset{y}{\sum }exp\left(\overset{n}{\underset{i=1}{\sum }}w_{i}f_{i}\left(x,y\right)\right)$$

where x represents each input environmental variable, y denotes the locations of the grasshopper occurrence, $f_{i}\left(x,y\right)$ is the characteristic function, $w_{i}$ is the weight of the characteristic function, n represents the number of datasets, and $P_{w}\left(y|x\right)$ is the output of the spatial distribution of the grasshopper occurrence in two grass types.

The ‘subsample’ routine was used for the Maxent model with 30 replicate runs. A total of 70% of the randomly selected grasshopper occurrence data were used for model training and 30% for model testing in each run. The convergence threshold was set to 0.5. The omission curve and ROC curve were used to evaluate the accuracy of the model. The value of the area under the curve (AUC) in the ROC curve varies from 0.5 to 1, where 0.5 indicates a random model and a value close to 1 represents high discrimination. A higher AUC value indicates a higher efficiency of the models. In addition, the ‘Percent variable contributions’ were selected to detect the key environmental factors for grasshopper occurrence. The ‘Response Curve’ routine was used to examine the specific relationships between the key environmental factors and the probability of presence of grasshopper occurrence.

### 2.5. Analysis Framework

**Figure 3:**
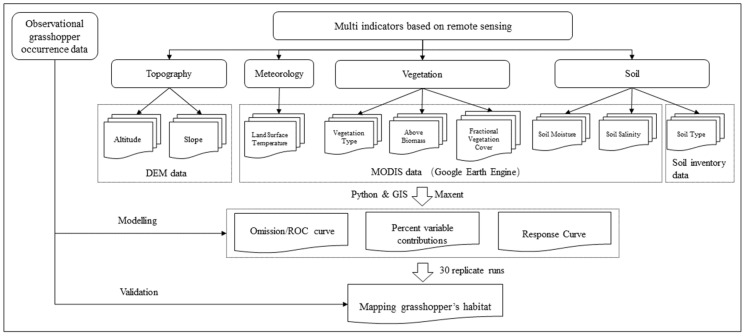
Flowchart of this study. Key factors of grasshopper occurrence in typical steppe and meadow steppe were detected by integrating machine learning model and remote sensing data. Observed grasshopper occurrence data are used for modelling and validation. Factors include topography (Altitude, Slope), meteorology (Land Surface Temperature), vegetation (Vegetation Type, Above Biomass, Fractional Vegetation Cover) and soil (Soil Type, Soil Moisture, Soil Salinity). Python, GIS and Maxent are used to achieve the modelling and validation.

The factors cased from remote sensing data that were used to model the suitability of grasshopper occurrence were first collected from DEM data, soil inventory data and MODIS data (cf. Figure 3). The MODIS data downloading and computation of related remote sensing indicators were performed by Google Earth Engine. A batch processing code based on Python in ArcGIS was then used to pre-process to a unified data coordinate system, spatial resolution and data analysis range. All the environmental variable data used in this study were either at 1 km resolution or resampled to 1 km resolution using the nearest-neighbor method. To avoid strong collinearity between variables, variables with Pearson correlation coefficients less than 0.9 were retained. The statistical analysis was implemented in SPSS Statistics 22.0 (IBM Corp, Armonk, NY, USA). Afterwards, by integrating the multi-indicators based on remote sensing and observational grasshopper occurrence data, the Maxent model was used for mapping the grasshopper occurrence area and analyzing the key relevant environmental factors through the omission/ROC curve, percent variable contributions and response curve.

## 3. Results

### 3.1. The Spatial Distribution of Grasshopper Occurrence in Typical Steppe and Meadow Steppe

**Figure 4:**
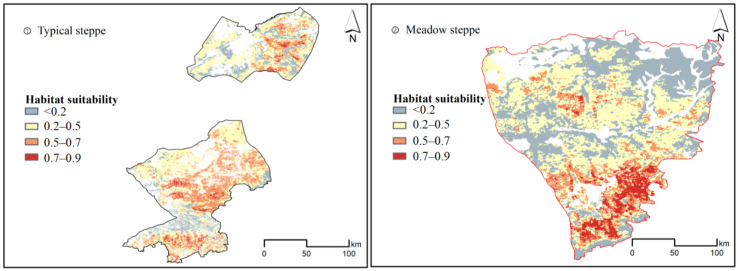
Modelling suitability of grasshopper occurrence in typical and meadow steppes by Maxent models. The suitability degrees of grasshopper occurrence are classified into four levels: high suitability (>0.7, red color), moderate suitability (0.5–0.7, orange color), low suitability (0.2–0.5, yellow color), and unsuitable (<0.2, gray color).

The Maxent modelling results for suitability of grasshopper occurrence in typical steppe and meadow steppe are shown in Figure 4. The suitability degree of grasshopper occurrence is classified into four levels: high suitability (>0.7), moderate suitability (0.5–0.7), low suitability (0.2–0.5), and unsuitable (<0.2). In the typical steppe, 2% of the grassland has a high suitability for grasshopper occurrence while 22.6% has a moderate suitability. In the meadow steppe, 5.7% of the grassland has a high suitability of grasshopper occurrence while 12.9% has a moderate suitability (cf. Table 2). Totally, considering the sum of moderate and highly suitable areas, the typical steppe has a larger area and a greater proportion of suitable area for grasshopper occurrence (with 24.6% of whole grassland) than the meadow steppe (with 18.6% of whole grassland).

**Figure 5:**
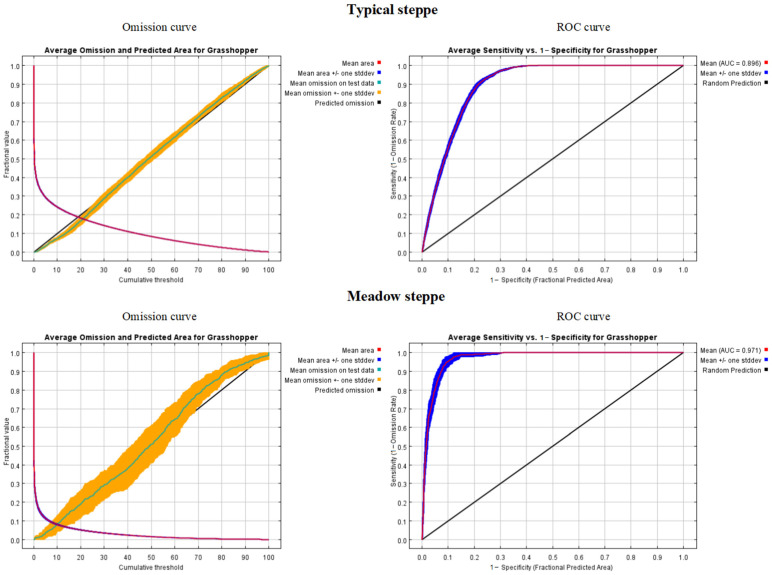
Omission curves and ROC curves in Maxent models for typical and meadow steppes. The omission curve shows how testing and training omission and predicted area vary with the choice of cumulative threshold (suitable conditions predicted above the threshold and unsuitable below). Normally, the omission rate should be close to the predicted omission according to the definition of the cumulative threshold. The receiver operating characteristic (ROC) curve includes a red line and blue line and an AUC value. The red (training) line shows the fit of the model to the training data. The blue (testing) line indicates the fit of the model to the testing data.

Through validation based on 30% of the investigative grasshopper occurrence data, the omission curve and ROC curve show the modelling accuracy for grasshopper occurrence in typical steppe and meadow steppe (cf. Figure 5). The omission curves of typical steppe and meadow steppe both show that the mean omission on test samples is close to the predicted omission (the black straight line), which indicates a good fit between the model and the training data, and that the test data and training data are independent. The AUC curve shows that in typical steppe and meadow steppe, the value of the simulation result is greater than that of the random model (0.5), which indicates that the simulated distribution of grasshopper occurrence is consistent with the collected data. The modelling accuracy in meadow steppe is better than that in typical steppe. The efficiency for modeling grasshopper occurrence in meadow steppe (AUC = 0.971) is better than the efficiency for typical steppe (AUC = 0.896).

### 3.2. The Key Factors of Grasshopper Occurrence in Typical Steppe and Meadow Steppe

Estimates of relative percentage contributions of all the environmental variables (cf. Table 3) show that the soil type, above biomass in the eclosion period, vegetation cover in the nymph period, altitude and minimum LST in the incubation period have the most predictive power for modeling grasshopper occurrence in typical steppe, with 21.9%, 17.5%, 17%, 12.5% and 11.8% contributions, respectively. These environmental factors can totally explain 80.7% of the distribution of grasshopper occurrence in typical steppe.

The above biomass in the nymph period, and altitude, soil type and the minimum LST in the overwintering period have the most predictive power for modeling grasshopper occurrence in meadow steppe, with 33%, 29.6%, 9.8% and 8.7% contributions, respectively. These environmental factors can explain 81.1% of the distribution of grasshopper occurrence in meadow steppe.

In summary, the soil type, above biomass, altitude and temperature mainly determine the suitability for grasshopper occurrence in both typical steppe and meadow steppe. The vegetation cover also determines the grasshopper occurrence in typical steppe.

### 3.3. The Role of Top Contributing Factors for Grasshopper Occurrence in Typical Steppe and Meadow Steppe

Individual response curves describing the relationships between grasshopper occurrence and the top contributing factors in typical steppe and meadow steppe are shown in Figure S1. The response curve for the model in typical steppe shows that the main soil types with high suitability for grasshopper occurrence are Kastanozems, Castano–cinnamon soils and Skeletol soils in order. In addition, the suitability for grasshopper occurrence has a relatively high value when the above biomass in the eclosion period is between 100 kg/hm^3^ and 175 kg/hm^3^ and has the highest value when the above biomass is 140 kg/hm^3^. Moreover, the suitability has a high value when the vegetation cover in nymph period is between 0.22 and 0.45 and has the highest value when the vegetation cover is 0.28. Furthermore, the suitability has a high value when the altitude ranges between 650 and 700 m and 1250 and 1450 m and has the highest value when the altitude is 1400 m. The suitability of grasshopper occurrence has a high value when the minimum LST in incubation period ranges from −3.5 to −0.5 °C and has the highest value when the minimum LST is −1.9 °C.

The response curve for the model in meadow steppe shows that the suitability of grasshopper occurrence has a high value when the above biomass in the nymph period is between 135 kg/hm^3^ and 190 kg/hm^3^ and has the highest value when the above biomass is 145 kg/hm^3^. The suitability is high when the altitude is between 900 m and 1100 m and has the highest value when the altitude is 950 m. The main soil types with high suitability are Castano–cinnamon soils, Skeletol soils and Bog soils in order. The suitability has a high value when the minimum LST in the overwintering period is between −25 °C and −19 °C and has the highest value when the minimum LST is between −23.5 °C and −22.5 °C.

## 4. Discussion

### 4.1. Environmental Factors Interpreting the Differences between Typical Steppe and Meadow Steppe

Inner Mongolia of China has vast grassland covering different climate types with spatial heterogeneity in temperature and precipitation, large fluctuations in topography, soil status and vegetation status. Therefore, not all the areas are suitable for the growth of grasshoppers and the suitable environments for grasshoppers in diverse grass types are different. As shown from the response curves of the top predictor environmental variables in the grasshopper occurrence for typical steppe and meadow steppe (Figure S1), the difference in habitat range or niche range of grasshoppers could partly explain that typical steppe has a larger suitable area and greater proportion of suitable habitat for grasshopper occurrence than meadow steppe. In terms of soil type, altitude and above biomass, the habitat range of grasshoppers in typical steppe is higher than that in meadow steppe.

Previous studies have mainly discussed the impacts of meteorological factors on grasshopper occurrence [31,32,33,34]. This study confirmed that the meteorological factor (minimum land surface temperature) greatly influenced grasshopper occurrence. However, other environmental factors such as the topography, vegetation and soil conditions also played a role according to the data in this study.

Compared with other studies [54], this study not only constructed an index system and models for grasshopper occurrence suitability, but also analyzed and compared the similarities and differences in the environmental conditions. We comprehensively considered 16 environmental variables corresponding to four categories: topography, meteorology, vegetation and soil, in order to determine the key factors that impact grasshopper occurrence in typical steppe and meadow steppe. The results showed that 7 of the 16 environmental factors were strongly associated with the spatial distribution of grasshopper occurrence. All these environmental factors composed a specific range of habitat preferences for the grasshoppers. Although the soil type, above biomass, altitude and temperature were the main factors determining grasshopper occurrence in typical steppe and meadow steppe, the contribution orders of these factors in the two grass types were clearly different. In typical steppe, the soil type, above biomass in the eclosion period and vegetation cover in the nymphal period were the most important factors. In meadow steppe, the above biomass in the nymphal period and altitude were the most significant factors. The meteorological factors, especially the temperature, were also important in determining grasshopper occurrence in the two grass types.

Moreover, this study indicated that grasshopper occurrence in typical and meadow steppe were affected by vegetation and meteorological factors in different growth stages. The grasshopper populations in typical steppe were more influenced by above biomass in eclosion period and vegetation cover in the nymph period. However, the grasshopper populations in meadow steppe were more influenced by above biomass in nymph. The grasshopper occurrence in typical steppe was more affected by minimum land surface temperature in the incubation period. However, grasshopper occurrence in meadow steppe was more affected by minimum land surface temperature in the overwintering period.

### 4.2. Efficiency of Combining the Maxent Model and Remote Sensing Data

In this study, the Maxent model and remote sensing data were used to conduct the mapping of grasshopper occurrence and to identify the key environmental factors influencing grasshopper occurrence within two different grass types. Facing the difficulties of monitoring and prediction of grasshopper occurrence at a large scale [7], this study showed that the remote sensing data provided significant contributions to modeling and explaining the spatial distribution of grasshopper occurrence by expanding the spatial scale and directly providing related environmental data. This study used the remote sensing data to obtain the environmental variables including vegetation status and meteorological status during the main growth periods of grasshoppers. Furthermore, remote sensing data also provided the possibility of real-time monitoring and short-term prediction of grasshopper occurrence, and collectively improved the understanding of the key indicators of grasshopper occurrence. The improved spatial resolution of satellite observations can be helpful to the identification of grasshopper occurrence [7]. Remote sensing data will be more efficient in improving the monitoring of grasshopper pests in the future.

The machine learning model has the advantages of being fast, efficient and uses self-learning to simulate and analyze the effects of multiple factors on grasshopper occurrence [55,56]. The tests of the Maxent models in this study showed that the modelling results derived from remote sensing data have high accuracy and efficiency comparing with other ecological niche modeling (ENM) models such as GARP and BIOCLIM [57]. The combination of Maxent models and remote sensing data can provide a reasonable first approximation of the environmental factors affecting grasshopper occurrence [58]. This study also provided a methodological support for early warning and efficient prevention of grasshopper outbreaks. Furthermore, the species–environmental matching model could be tried with selecting more environmental indicators to deeply analyze the relationship between environmental factors and grasshopper occurrence. In addition, in the simulation, attention should be paid to increasing the amount and improving the accuracy of grasshopper occurrence data.

### 4.3. Management Implications

Grasshoppers have always been the most significant grassland pests in China [2,54]. Grasshoppers have caused widespread agricultural and social economic damage [5,6,54]. In this paper, the machine learning model (Maxent) and remote sensing data were used to develop a species–environmental matching model, study the spatial distribution of grasshopper occurrence in the major grasshopper occurrence area of Inner Mongolia, identify the related environmental variables, and summarize the suitable environmental conditions for grasshopper occurrence. This study provided a methodological support for early warning, efficient prevention and control of grasshopper populations. It also provided a decision basis for future management measures. The proposed method can be further used in the selection of priority control sites [56]. Moreover, clearly defining different key environmental factors that influence grasshopper occurrence in typical steppe and meadow steppe will help to provide a priori knowledge for early control. It will also help to predict the dynamics of grasshopper occurrence when environmental factors change in different grassland types, and to guide the management to areas where potential grasshopper occurrence is the highest [56].

## 5. Conclusions

This paper studied the spatial distribution and key factors of grasshopper occurrence in two grass types. Grasshopper occurrence records from 2020, machine learning models (Maxent), and remote sensing data were used to develop a species–environmental matching model, identify grasshopper occurrence in the major grasshopper occurrence area of Inner Mongolia, analyze the related environmental variables (including topography, meteorology, vegetation, and soil), and summarize the key factors and suitable environment conditions for grasshopper occurrence in typical steppe and meadow steppe. The results of this study showed that the typical steppe had a larger suitable area and greater proportion of suitable habitat for grasshopper occurrence than meadow steppe. In summary, the soil type, above biomass, altitude, and temperature determined the suitability for grasshopper occurrence in typical steppe and meadow steppe. The amount of vegetation covers also determined grasshopper occurrence in typical steppe. All these environmental factors composed a range of habitat preferences for the grasshoppers. However, the contribution orders of these factors in the two grass types were clearly different. In addition, the vegetation and meteorological factors affected the grasshopper occurrence in different grasshopper growing stages. This study provided a methodological support for the early warning of grasshopper outbreaks and clearly defined the different key environmental factors of grasshopper occurrence in typical steppe and meadow steppe. It also provided a decision basis for future management measures.

## Figures and Tables

**Table 1:** Environmental variables used in this study that may influence grasshopper occurrence in grasslands.

Category	Environmental Variables	Spatial Resolution	Data Content and Source
Topography	Altitude	90 m	(Geospatial Data Cloud, http://www.gscloud.cn/, accessed on 5 November 2020)
	Slope	90 m	
Meteorology	Land Surface Temperature	1 km	Minimum LST: Spawning period (August–October 2019), Overwintering period (November 2019–March 2020), Incubation period (April–May 2020) Mean LST: Incubation period (April–May 2020) (MOD11A2.006 Terra Land Surface Temperature and Emissivity 8-Day Global 1 km, https://lpdaac.usgs.gov/products/mod11a2v006/, accessed on 20 June 2021)
Vegetation	Vegetation Type	1 km	(Resource and Environment Science and Data Center, https://www.resdc.cn/, accessed on 11 July 2019)
	Above Biomass	1 km	Nymph period (June 2020), Eclosion period (July 2020) (AB = 26.38e (3.8725 × NDVI), NDVI data derived from MOD13A2.006 Terra Vegetation Indices 16-Day Global 1 km, https://lpdaac.usgs.gov/products/mod13a2v006/, accessed on 20 June 2021)
	Fractional Vegetation Cover	1 km	Nymph period (June 2020), Eclosion period (July 2020) (FVC = (NDVI − NDVI_soil_)/(NDVI_veg_ − NDVI_soil_), NDVI data derived from MOD13A2.006 Terra Vegetation Indices 16-Day Global 1 km, https://lpdaac.usgs.gov/products/mod13a2v006/, accessed on 20 June 2021)
Soil	Soil Type	1 km	(Resource and Environment Science and Data Center, https://www.resdc.cn/, accessed on 1 April 2019)
	Soil Moisture Index	1 km	Spawning period (August–October 2019), Incubation period (April–May 2020) (TVDI = (Ts − Ts min)/(Ts max − Ts min), Ts max = a × NDVI + b, Ts min = c × NDVI + d, LST data derived from MOD11A2.006 Terra Land Surface Temperature and Emissivity 8-Day Global 1 km https://lpdaac.usgs.gov/products/mod11a2v006/, accessed on 20 June 2021, NDVI data derived from MOD13A2.006 Terra Vegetation Indices 16-Day Global 1 km https://lpdaac.usgs.gov/products/mod13a2v006/, accessed on 20 June 2021)
	Soil Salinity Index	1 km	Spawning period (August–October 2019), Incubation period (April–May 2020) $(\mathrm{SI}=\sqrt{\mathrm{Bg}\;*\;\mathrm{Br}}$, Bg and Br are the reflectance in the green band and red band that are derived from MOD09A1.006 Terra Surface Reflectance 8-Day Global 500 m, https://lpdaac.usgs.gov/products/mod09a1v006/, accessed on 20 June 2021)

**Table 2:** Proportion of each suitability level in typical steppe and meadow steppe.

Level of Suitability	Typical Steppe	Meadow Steppe
Unsuitable	37.4%	40.0%
Low	38.1%	41.5%
Moderate	22.6%	12.9%
High	2.0%	5.7%

**Table 3:** Relative contributions of the environmental variables to grasshopper occurrence.

Typical Steppe		Meadow Steppe	
Environmental Variables	Percentage Contribution	Environmental Variables	Percentage Contribution
Soil type	21.9%	Abio_Nymph	33%
Abio_Eclosion	17.5%	Altitude	29.6%
FVC_Nymph	17%	Soil type	9.8%
Altitude	12.5%	Lstmin_Overwintering	8.7%
Lstmin_Incubation	11.8%		
Total	80.7%	Total	81.1%

## Data Availability

The data are not publicly available because the data needs to be used in future work.

## Supplementary Materials

The following supporting information can be downloaded at https://www.mdpi.com/article/10.3390/insects13100894/s1, Figure S1: Response curves of the top predictor environmental variables in the grasshopper occurrence for typical steppe (①) and meadow steppe (②). Abscissa axis represents the range of variables, and ordinate axis shows the suitability. Top variables for typical steppe include soil type, above biomass in eclosion period, fractional vegetation cover in nymph period, altitude and minimum land surface temperature in incubation period, and for meadow steppe include above biomass in nymph period, altitude, soil type and minimum land surface temperature in overwintering period.
