# Supplementary material for: Detecting Key Factors of Grasshopper Occurrence in Typical Steppe and Meadow Steppe by Integrating Machine Learning Model and Remote Sensing Data
Source: Insects. 2022 Sep 30;13(10):894. doi: 10.3390/insects13100894 (PMC9603866; doi:10.3390/insects13100894)
Supplement: Supplementary file 1 [file insects-13-00894-s001.zip › insects-1900996 - supplementary.pdf]

insects

MDPI

① Typical steppe

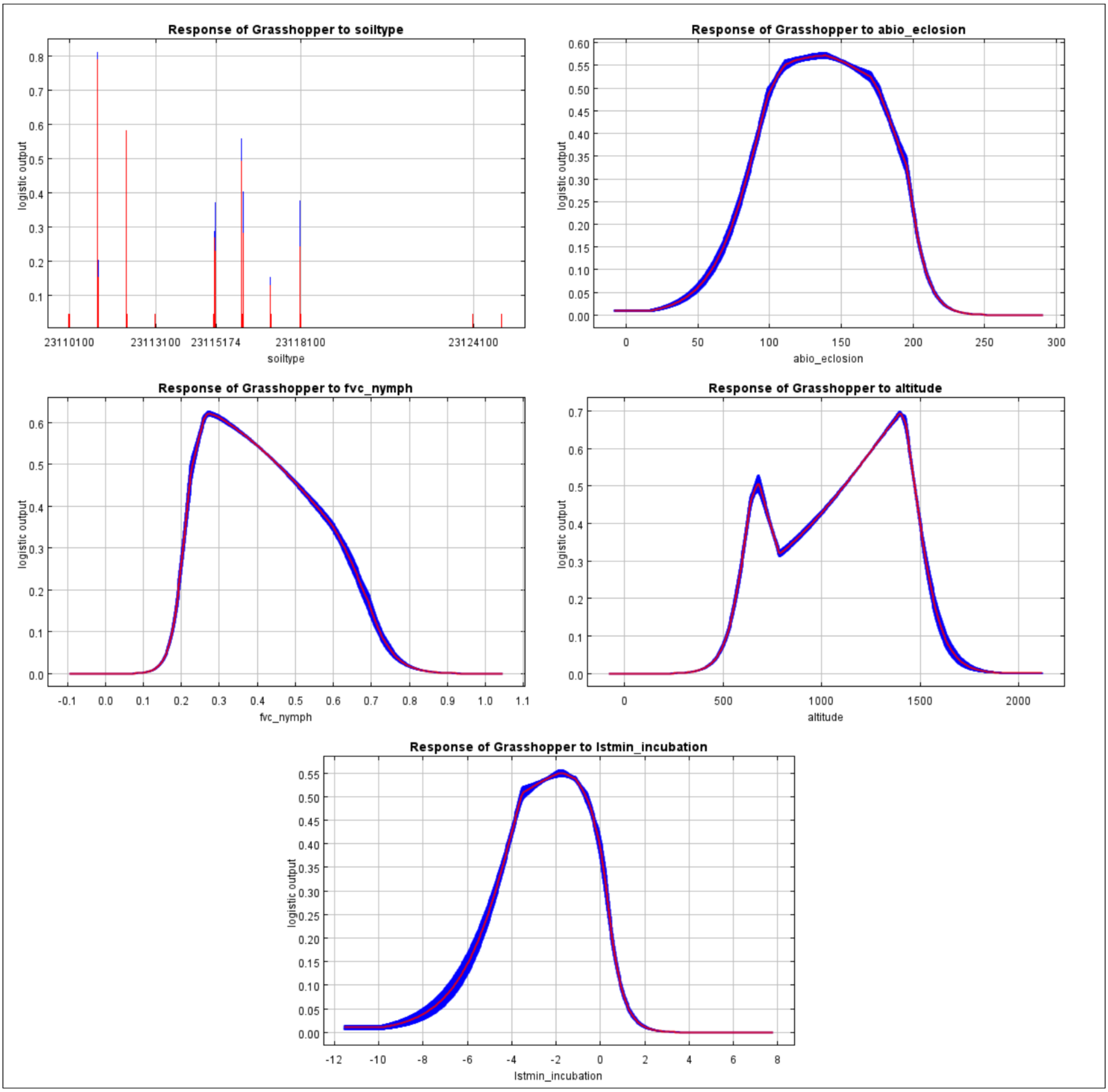

② Meadow steppe

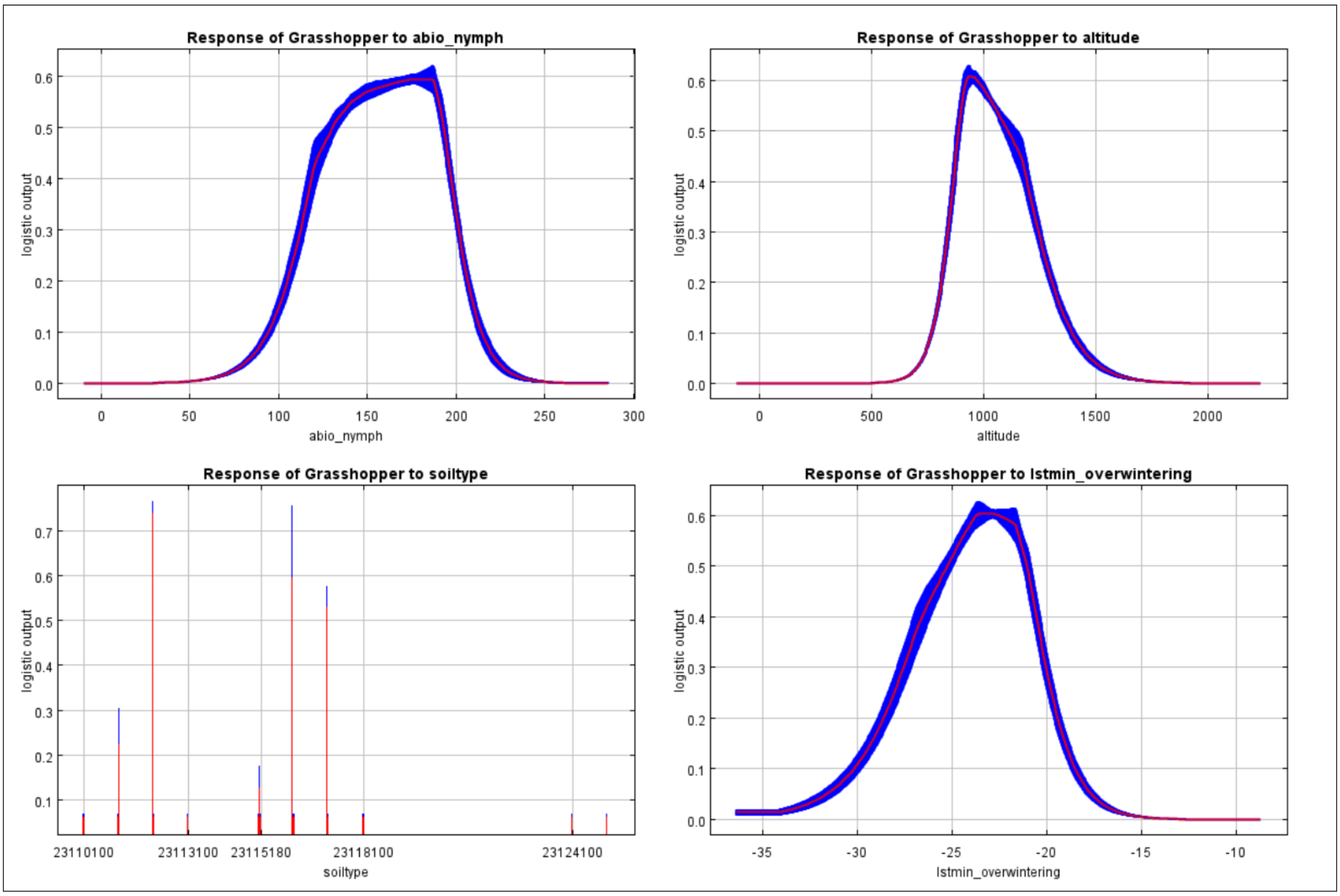

Figure S1. Response curves of the top predictor environmental variables in the grasshopper occurrence for typical steppe (①) and meadow steppe (②). Abscissa axis represents the range of variables, and ordinate axis shows the suitability. Top variables for typical steppe include soil type, above biomass in eclosion period, fractional vegetation cover in nymph period, altitude and minimum land surface temperature in incubation period, and for meadow steppe include above biomass in nymph period, altitude, soil type and minimum land surface temperature in overwintering period. Notes: The codes of soil type are as follows: Brown coniferous forest soil (23110100), Castanozems (23112108), Castano-cinnamon soils (23112112), Brown calcic soil (23113100), Purple soil (23115174), Chisley soil (23115180), Skeletol soils (23115194), Bog soils (23117100), Saline soil (23118100), Lake reservoir (23124100).
